# A network meta-analysis of curative effect of different treatment methods on patients with brain metastasis of breast cancer

**DOI:** 10.1097/MD.0000000000030392

**Published:** 2022-09-02

**Authors:** Anhao Wu, Fang Zhang, Xin Yang, Yang Liu, Mingjian Tan, Yafang Lai, Zhuangqing Yang

**Affiliations:** a Department of Mammary Surgery I, The Third Affiliated Hospital of Kunming Medical University (Yunnan Cancer Hospital, Yunnan Cancer Center), Kunming, China; b Department of Tumor 2 Families, Central Hospital of Enshi Tujia and Miao Autonomous Prefecture, Enshi, China; c Department of Blood Transfusion, The First People’s Hospital of Yunnan Province, The Affiliated Hospital of Kunming University of Science and Technology, Kunming, China; d Kunming women and Child Health Service Center/Kunming women and Child Health Care Hospital, Kunming, China.

**Keywords:** brain metastasis, breast cancer, network meta-analysis, treatment measures

## Abstract

Breast cancer (BC) is a malignant tumor with the highest incidence rate worldwide, and its incidence of breast cancer brain metastases is increased in recent years. Although significant progress has been made in the systematic treatment of BC that of breast cancer brain metastases is still very difficult. Organically integrating local and systemic therapies remains an urgent problem to be solved. In this study, a network meta-analysis was performed to collect the treatment effects of different treatment measures on patients with BC brain metastasis in recent years, evaluate and screen the current best clinical treatment scheme, and assist doctors in formulating clinical treatment schemes.

**Methods:**

Keywords were used to search databases, such as the Chinese Journal Full-text Database, VIP Chinese Science and Technology Journal Full-text Database (VP-CSJFD), Wanfang Data Journal Paper Resources (Wangfang), PubMed, the Cochrane Library, and EMBASE. The retrieval period was from the establishment of each database to February 2022. Qualified randomized controlled studies were screened according to the inclusion and exclusion criteria, and Stata 16 software was adopted for mesh meta-analysis of binary variable data. Using R4 0.2 software, and calling GeMTC and JAGS packages in R software, the Bayesian network model analysis of survival data was completed.

**Conclusion:**

Combined with overall response rate, disease control rate, and overall survival, whole-brain radiation therapy + 3-dimensional conformal radiation therapy + Che may be the intervention measure with the highest objective remission rate for patients with brain metastasis of BC, besides, it may also be the intervention measure of the highest disease control rate in patients after treatment. In contrast, WBRT + Che may be the intervention with the lowest overall survival risk ratio after treatment.

## 1. Introduction

Breast cancer (BC) is a malignant tumor with the highest incidence rate in the world,^[1]^ and 10% to 20% of patients with advanced BC will develop brain metastasis.^[2,3]^ They have poor treatment effects, rapid progression, and high mortality, with a survival time of only 2 to 25.3 months.^[4]^ In recent years, with the rapid development of systemic therapy, extracranial lesions of BC have been effectively controlled, the survival time of patients has been prolonged, and the possibility of brain metastasis is also increasing. In addition, advances in imaging technology and the popularization of conventional imaging monitoring have increased the detection rate of intracranial lesions. Considering this, brain metastasis of BC has become increasingly common in clinical practice. However, many chemotherapeutic drugs cannot penetrate the blood-brain barrier. In this case, the survival time of patients with brain metastasis was shorter, but treatment could be selected. At present, treatment methods mainly include surgery, stereotactic radiotherapy, and whole-brain radiotherapy (WBRT).^[5–7]^ WBRT refers to radiotherapy of the whole brain to control the growth of intracranial tumors. Stereotactic radiation treatment (SRT) is to use special equipment realize focused radiotherapy with small irradiation field through stereotactic and positioning technology. Three-dimensional conformal radiation therapy (3D-CRT) is to make the geometry of the irradiation field consistent with the shape of the tumor in the 3D direction. In this study, a network meta-analysis was performed to collect the treatment effects of different treatment measures on patients with BC brain metastasis in recent years, evaluate and screen the current best clinical treatment scheme, and assist doctors in formulating clinical treatment schemes.

## 2. Data and methods

### 2.1. Data

Keywords were employed to search databases such as the Chinese Journal Full-text Database, VIP Chinese Science and Technology Journal Full-text Database (VP-CSJFD), Wanfang Data Journal Paper Resources (Wangfang), PubMed, Cochrane Library, and EMBASE. The retrieval period was from the establishment of each database to February 2022. The retrieval formulas adopted were as follows: whole-brain radiotherapy OR radiosurger OR gamma knife radiosurgery OR stereotactic radiation OR stereotactic radiosurgery OR LINAC radiosurgery OR linear accelerator radiosurgery OR (stereotactic body radiotherapy OR CyberKnife radiosurgery OR stereotactic radiation therapy OR surgery OR operative therapy OR invasive procedures OR operative procedures OR operations AND Breast Neoplasms OR Breast Neoplasm OR Breast Tumor OR Mammary Cancer OR Malignant Neoplasm of Breast OR Breast Malignant Neoplasm OR Malignant Tumor of Breast OR Breast Malignant Tumor OR Cancer of the Breast OR Human Mammary Carcinoma OR Human Mammary Neoplasm OR Breast Carcinoma AND Brain OR Encephalon AND metastasis.

The inclusion criteria were as follows: (1) the subjects included in the literature were definitively diagnosed with brain metastasis of BC; (2) the document language was limited to Chinese and English; (3) the age, race, course of disease, and pathological type of BC in the literature were not limited; (4) the research focused on stereotactic radiotherapy and stereotactic radiotherapy; and (5) the outcome indicators of the study included overall survival (OS), objective remission rate, and DCR.

Exclusion criteria: (1) repeated publications without finding the original text; (2) overview, experience summary, case report, meeting, meta-analysis, etc; (3) correctly diagnosed brain metastases of BC; and (4) intervention measures excluding whole-brain radiotherapy, stereotactic radiotherapy, and surgical treatment.

### 2.2. Protocol registration

This system review program strictly follows the system review and meta-analysis program (PRISMA-P) preferred reporting items.^[8]^ The system review program was registered on the INPLASY website (registration number: INPLASY202250054). If adjustments are made during the entire study period, we will fix and update the detailed information in the final report on time.

### 2.3. Method

(1) Import the retrieved literature into endnote software, delete the duplicate literature after duplicate checking, and eliminate the articles that do not meet the requirements by reading the titles and abstracts of the remaining literature according to the above-mentioned inclusion and exclusion criteria. (2) For the remaining literature, 2 researchers read the full text in detail, and the literature excluding outcome indicators, incomplete data, or repeated data results will be deleted. (3) To ensure the accuracy of the data and the precision of the research, the researchers extracted the relevant data. The extracted content mainly included the research author, publication year, baseline, intervention measures, and outcome indicators. After data were extracted and improved, they were integrated and checked. In the case of disagreement in the above process, a third-party expert with many years of experience in evidence-based medicine shall be invited to make a joint judgment that shall be the final result.

Two researchers adopted the Newcastle Ottawa scale, which consists of selection (4 items), comparability (1 item), and outcome (3 items) to evaluate the quality of the included cohort studies. The highest score for each item of selection and outcome was 1, while that of comparable items was 2, and the total score of the evaluation result of the scale was 9. A score (0–4) means low-quality literature, and (5–9) refers to high-quality literature. In addition, the Jadad scale was used to evaluate the quality of the included randomized controlled studies when the evaluation content included random grouping, allocation concealment, blind methods, and the description of loss of follow-up and withdrawal. A score (0–3) is classified as low-quality literature, while (4–7) belong to high-quality literature.

### 2.4. Statistical methods

Stata 16 software was adopted for the network meta-analysis of binary variable data, while an inconsistency test was conducted to analyze the overall consistency between direct and indirect evidence. When *P* > .05, there was no consistency and the consistency model was fitted. In contrast, an inconsistent model was fitted. In addition, the node-splitting method was used to test the local inconsistency between the direct and indirect comparisons. When *P* < .05, local inconsistencies were observed. The count data are expressed as relative risk (HR) and 95% confidence interval (CI). Furthermore, the efficacy of intervention measures is ranked according to the area under the cumulative probability (surface under the cumulative ranking, SUCRA). The larger the area under the curve, the better is the efficacy of the intervention measures.

Using R4.0.2 software, we call the packages of GeMTC and JAGS in R software to complete the Bayesian network model analysis of survival data. Consistent deviance information criteria (DIC) value and inconsistent DIC value are used to test and analyze the overall consistency between direct evidence and indirect evidence. If the difference of DIC between the 2 is <5, it is considered that there is no consistency, and the consistency model is fitted, otherwise the inconsistency model is fitted. The node-splitting method was used for direct and indirect comparison of local inconsistencies. *P* < .05 showed that there was local inconsistencies. Survival data were expressed by risk ratio (HR) and its 95% CI. The drugs with the best curative effect are judged according to the ranking probability of intervention drugs obtained under the Bayesian Network Model. The greater the probability, the better the survival and prognosis of patients.

When the number of studies included in the outcome index exceeded 10, publication bias was evaluated by visually observing the distribution symmetry of points on the funnel chart.

### 2.5. Ethics and dissemination

Because this is a systematic review of the protocol and a network meta-analysis, all the data in this study are from published studies and do not involve patients, so there is no need for ethical recognition. The results of this study will be distributed to peer reviews and presented at relevant meetings.

## 3. Results

### 3.1. Literature search results

**Figure 1.**
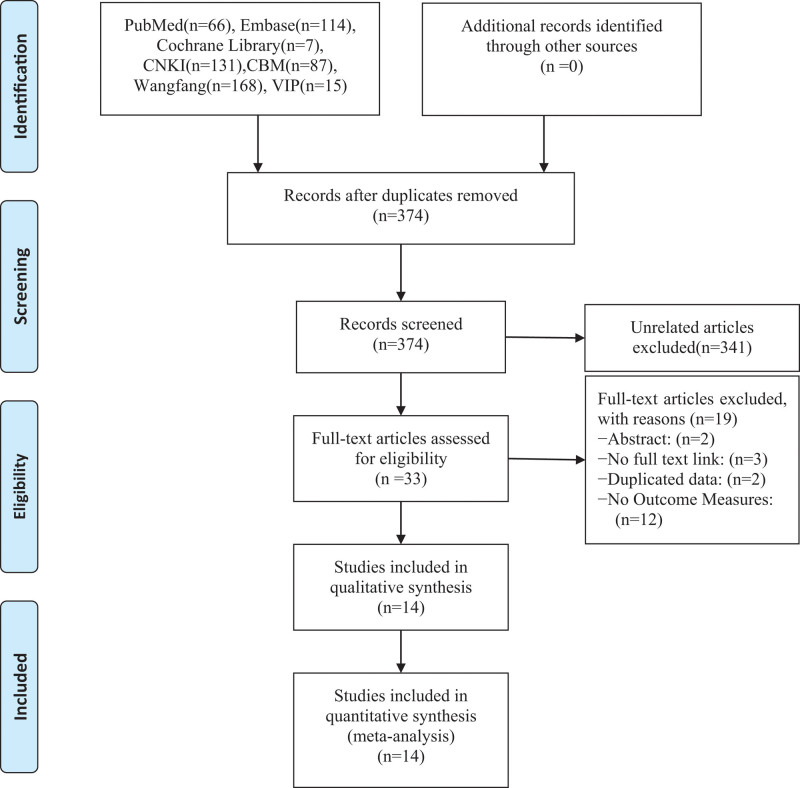
Flow chart of literature screening.

A total of 588 studies were obtained through a systematic search, and 374 were acquired after duplication using Endnote X9 software. After reading the title and abstract, 33 studies were obtained by excluding irrelevant studies, noncontrolled experimental studies, conferences, abstracts, meta-analyses, and others. For the remaining ones, after reading the full text, 14 were finally included,^[9–23]^ with a total of 1723 patients(see Fig. 1).

The basic information of the included studies is presented in Table 1. The overall quality of the included studies was acceptable in randomized controlled trials (Jadad score ≥ 4) and cohort studies (Newcastle Ottawa scale score ≥ 5).

**Table 1:** The basic characteristics and methodological quality assessment of included studies.

Author	Country	Year	Research type	Interventions		Sample size		Age (yr)		Types of Symptom	Outcome measures	Jadad/NOS score
				Observation group	Control group	Observation group	Control group	Observation group	Control group			
Bo Luo	China	2021	Cohort study	SRT + Che	WBRT + Che	42	49	<50 = 29 people, ≥50 = 13 people	<50 = 35 people, ≥50 = 14 people	BCBM	ORR, OS, DCR	5
Xinhai Bai	China	2019	Cohort study	WBRT + Che	WBRT	60	60	51.3 ± 3.2	50.4 ± 2.5	BCBM	ORR, DCR	4
Yun Zhang	China	2015	Cohort study	WBRT + 3D-CRT	3D-CRT	30	30	54.4 ± 1.6	54.3 ± 1.7	BCBM	ORR, DCR	4
Hanguang Ruan	China	2020	Cohort study	WBRT	WBRT + Che	25	21	<35 = 11 people, ≥50 = 14 people	<35 = 9 people, ≥50 = 12 people	BCBM	ORR, OS, DCR	5
Ying Mao	China	2016	Cohort study	WBRT	WBRT + Che	30	30	55 (29~75)		BCBM	ORR, DCR	5
Mei Yang	China	2018	Cohort study	WBRT	WBRT + Che	14	16	NA	NA	BCBM	ORR, DCR	5
Xiulong Zhang	China	2017	Cohort study	WBRT + 3D-CRT + Che	WBRT + 3D-CRT	44	48	48.7 ± 5.2	50.1 ± 4.6	BCBM	ORR, OS, DCR	5
Li Liu	China	2011	Cohort study	WBRT	WBRT + 3D-CRT	29	29	26~69		BCBM	ORR, DCR	5
K.I.Cao	France	2014	Cohort study	WBRT	WBRT + Che	50	50	57.8 (38~79)	53.6 (29~78)	BCBM	ORR, OS, DCR	6
Stephanie E. Combs	Germany	2004	Cohort study	SRT	WBRT + SRT	10	13	<40 = 19 people, ≥40 = 43 people		BCBM	OS	5
Anna Gullhaug	Norway	2021	Cohort study	WBRT	SRT	206	49	<65 = 178 people, ≥65 = 76people		BCBM	OS	6
Sung Sook Lee	Korea	2007	Cohort study	A = Operation, B = WBRT, C = No treatment		A = 29, B = 157, C = 9		45 (26–78)		BCBM	OS	5
Charles Scott	America	2007	Cohort study	WBRT	WBRT + Che	49	57	<65 = 39 people, ≥65 = 10 people	<65 = 46 people, ≥65 = 11 people	BCBM	OS	5
Joseph M Kim	America	2019	Cohort study	SRT	SRT + Che	355	132	52 (32~84)	50 (31~71)	BCBM	ORR, DCR	7

3D-CRT = 3-dimensional conformal radiation therapy, BCBM = breast cancer brain metastases, Che = chemotherapy, CI = confidence intervals, DCR = disease control rate, NOS = Newcastle Ottawa scale, ORR = overall response rate, OS = overall survival, SRT = stereotactic radiation treatment, WBRT = whole-brain radiation therapy.

### 3.2. Results of network meta-analysis

#### 3.2..1. Evidence network

**Figure 2.**
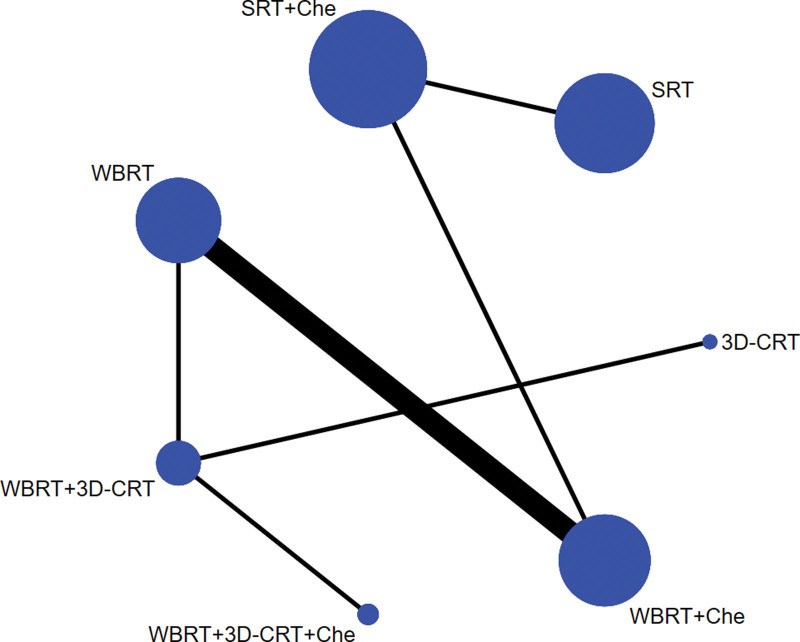
Network evidence map of ORR. ORR = overall response rate.

**Figure 3.**
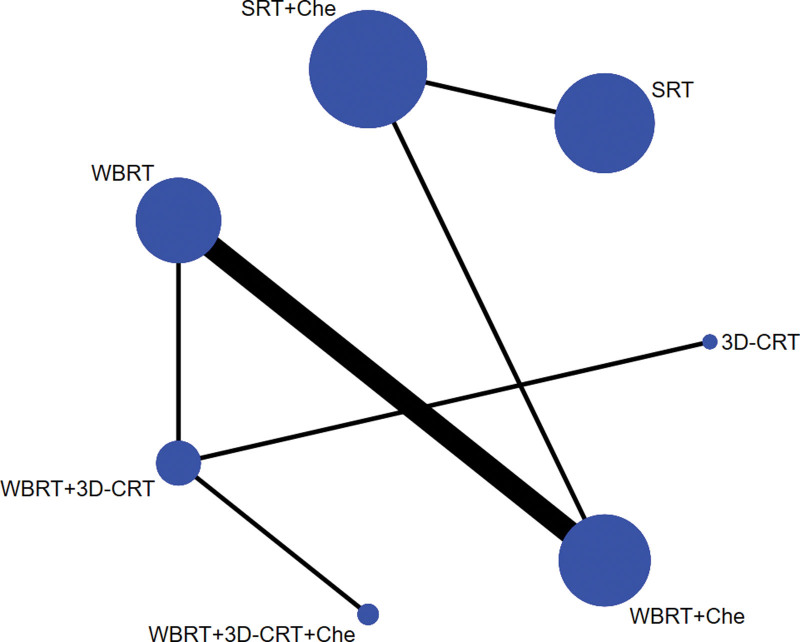
Network evidence map of DCR. DCR = disease control rate.

**Figure 4.**
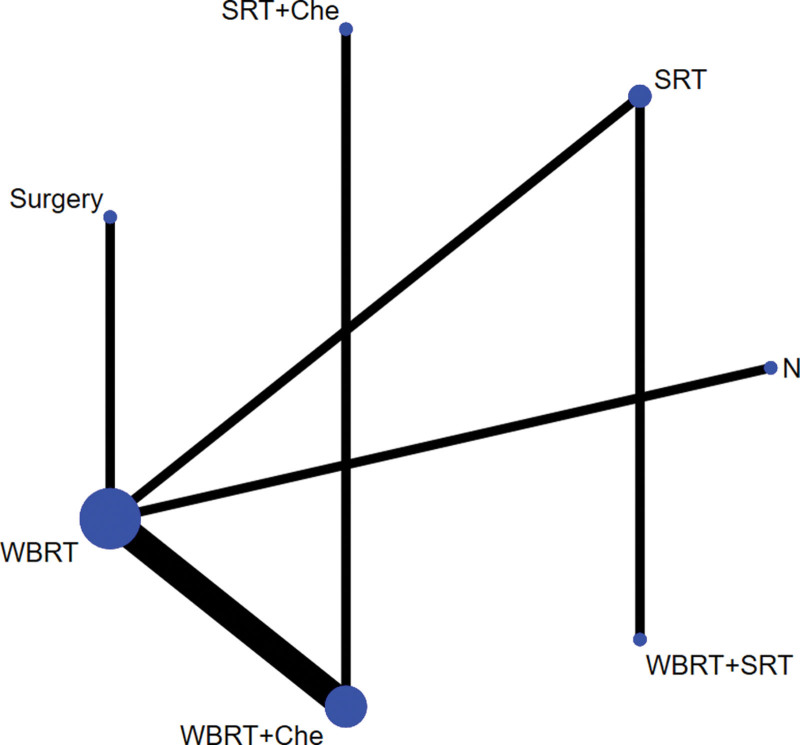
Network evidence map of OS. OS = overall survival.

Based on direct comparative data, a relationship between intervention methods was established. Each vertex of the graph represents different intervention methods: the vertex size refers to the sample size of each intervention method, and the line between the vertices indicates a direct comparison between the 2 intervention methods. In addition, the thickness of the line was directly proportional to the number of studies for each pair of intervention methods. There is direct or indirect evidence between different intervention methods, thus having the basic conditions for network meta-analysis (see Figs. 2 to 4).

#### 3.2..2. Consistency inspection

The model test results of inconsistency between the objective remission rate and disease control rate (DCR) showed *P* > .05, indicating that the overall inconsistency was not significant. The consistency DIC value of OS was 16.52, and the inconsistency DIC value was 16.53, with a difference of <5, implying that the overall inconsistency was not significant. Then, the node-splitting method was used to test for local inconsistency. The results showed that there was no inconsistency between the direct and indirect comparisons of objective remission rates and DCR (*P* > .05), and the local consistency was good. Therefore, consistency model analysis was performed.

#### 3.2..3. Overall response rate

Overall response rate were reported in 10 studies, and interventions included 3D-CRT, SRT, SRT + Che, WBRT, WBRT + 3D-CRT, WBRT + 3D-CRT + Che, and WBRT + Che. The results of the network meta-analysis showed that the objective remission rate of patients after WBRT treatment was lower than that of WBRT + 3D-CRT + Che, and the difference was statistically significant (RR = 0.22, 95% CI: 0.05–0.97, *P* < .05). In addition, the comparison between the objective remission rates of the other 2 interventions was not statistically significant (*P* > .05) (Table 2). Furthermore, the ranking results of the probability of each intervention becoming the best treatment measure showed that WBRT + 3D-CRT + Che (93.1%) > WBRT + 3D-CRT (59.5%) > SRT + Che (56.4%) > WBRT + Che (43.3%) > SRT (41.8%) > 3D-CRT (39.2%) > WBRT (16.5%), suggesting that WBRT + 3D-CRT + Che may be an intervention measure with the highest objective remission rate after treatment.

**Table 2:** Comparison results of network meta-analysis of ORR under various intervention measures (RR, 95% CI).

3D-CRT						
0.98 (0.17, 5.52)	SRT					
0.82 (0.18, 3.81)	0.84 (0.38, 1.84)	SRT + Che				
1.33 (0.38, 4.59)	1.35 (0.41, 4.51)	1.62 (0.65, 4.02)	WBRT			
0.75 (0.32, 1.76)	0.77 (0.17, 3.44)	0.92 (0.25, 3.29)	0.57 (0.23, 1.39)	WBRT + 3D-CRT		
0.29 (0.07, 1.25)	0.30 (0.04, 2.01)	0.36 (0.06, 2.03)	0.22 (0.05, 0.97)	0.39 (0.12, 1.26)	WBRT + 3D-CRT + Che	
0.98 (0.26, 3.65)	1.00 (0.33, 3.08)	1.20 (0.54, 2.67)	0.74 (0.48, 1.13)	1.31 (0.48, 3.54)	3.33 (0.72, 15.44)	WBRT + Che

3D-CRT = 3-dimensional conformal radiation therapy, Che = chemotherapy, CI = confidence intervals, DCR = disease control rate, NOS = Newcastle Ottawa scale, ORR = overall response rate, OS = overall survival, SRT = stereotactic radiation treatment, WBRT = whole-brain radiation therapy.

#### 3.2..4. Disease control rate

DCR was reported in 10 studies, and the interventions involved 3D-CRT, SRT, SRT + Che, WBRT, WBRT + 3D-CRT, WBRT + 3D-CRT + Che, and WBRT + C he. The results of the NMA showed that there was no statistical significance in the comparison of DCR between any 2 interventions (*P* > .05) (Table 3). Moreover, the ranking results of the probability of each intervention as the best treatment measure revealed that WBRT + 3D-CRT + Che (74.9%) > SRT + Che (59.7%) > WBRT + 3D-CRT (55.3%) > WBRT + C he (47.3%) > SRT (42.9%) > WBRT (39.1%) >3 D-CRT (30.7%), demonstrating that WBRT + 3D-CRT + Che may be an intervention measure of the highest DCR in patients after treatment.

**Table 3:** Comparison results of network meta-analysis of DCR under various intervention measures (RR, 95% CI).

3D-CRT						
0.92 (0.43, 2.01)	SRT					
0.85 (0.42, 1.69)	0.92 (0.64, 1.30)	SRT + Che				
0.93 (0.53, 1.63)	1.00 (0.59, 1.71)	1.10 (0.74, 1.64)	WBRT			
0.86 (0.58, 1.28)	0.93 (0.48, 1.80)	1.01 (0.58, 1.78)	0.92 (0.62, 1.38)	WBRT + 3D-CRT		
0.74 (0.42, 1.30)	0.80 (0.37, 1.75)	0.88 (0.44, 1.75)	0.80 (0.46, 1.41)	0.87 (0.58, 1.29)	WBRT + 3D-CRT + Che	
0.90 (0.50, 1.63)	0.97 (0.59, 1.60)	1.06 (0.75, 1.52)	0.97 (0.81, 1.17)	1.05 (0.68, 1.63)	1.21 (0.67, 2.19)	WBRT + Che

3D-CRT = 3-dimensional conformal radiation therapy, Che = chemotherapy, CI = confidence intervals, SRT = stereotactic radiation treatment, WBRT = whole-brain radiation therapy.

#### 3.2..5. Overall survival

OS was reported in 10 studies, and the interventions included 3D-CRT, SRT, SRT + Che, WBRT, WBRT + 3D-CRT, WBRT + 3D-CRT + Che, and WBRT + C he. The results of the NMA showed that there was no statistical significance in the comparison of OS between any 2 interventions (*P* > .05) (Table 4). In addition, the ranking results of the probability of each intervention as the best treatment measure revealed that WBRT + SRT (46.8%) > SRT + Che (18.2%) > SRT (14.9%) > Surgery (14.5%) > WBRT (2.1%) > Untreated (1.9%) > WBRT + Che (1.5%), indicating that WBRT + Che may be the intervention with the lowest risk ratio of OS after treatment.

**Table 4:** Comparison results of network meta-analysis of OS under various intervention measures (HR, 95% CI).

SRT						
0.71 (0.03, 14.29)	SRT + Che					
0.69 (0.04, 10.61)	0.96 (0.05, 20.82)	Surgery				
0.21 (0.01, 3.34)	0.29 (0.01, 6.79)	0.31 (0.02, 5.22)	Untreated			
0.6 (0.08, 4.1)	0.84 (0.09, 9.09)	0.87 (0.12, 6.4)	2.88 (0.38, 21.57)	WBRT		
0.49 (0.05, 4.51)	0.68 (0.09, 5.17)	0.71 (0.07, 6.68)	2.36 (0.22, 22.24)	0.81 (0.24, 2.47)	WBRT + Che	
1.54 (0.13, 17.47)	2.16 (0.05, 106.17)	2.21 (0.06, 88.14)	7.31 (0.18, 291.44)	2.59 (0.11, 56.29)	3.2 (0.12, 91.46)	WBRT + SRT

3D-CRT = 3-dimensional conformal radiation therapy, Che = chemotherapy, CI = confidence intervals, SRT = stereotactic radiation treatment, WBRT = whole-brain radiation therapy.

#### 3.2..6. Publication bias

**Figure 5.**
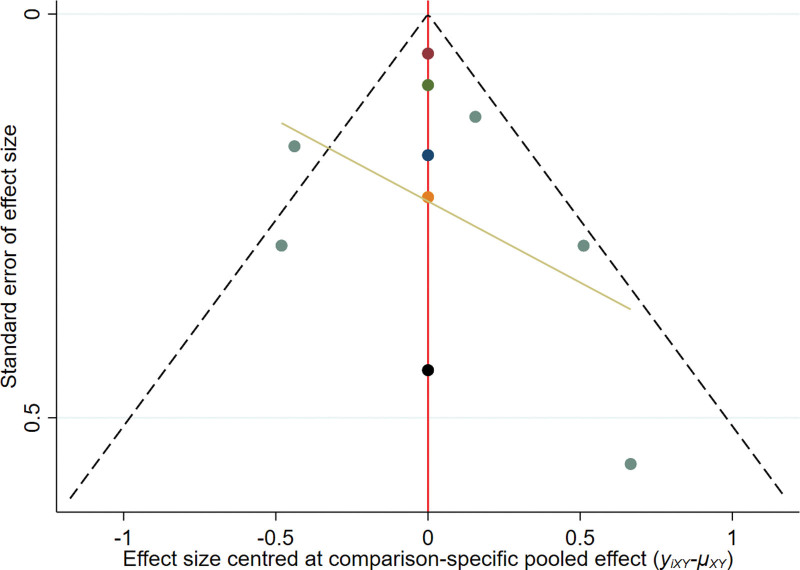
The funnel plot of ORR. ORR = overall response rate.

**Figure 6.**
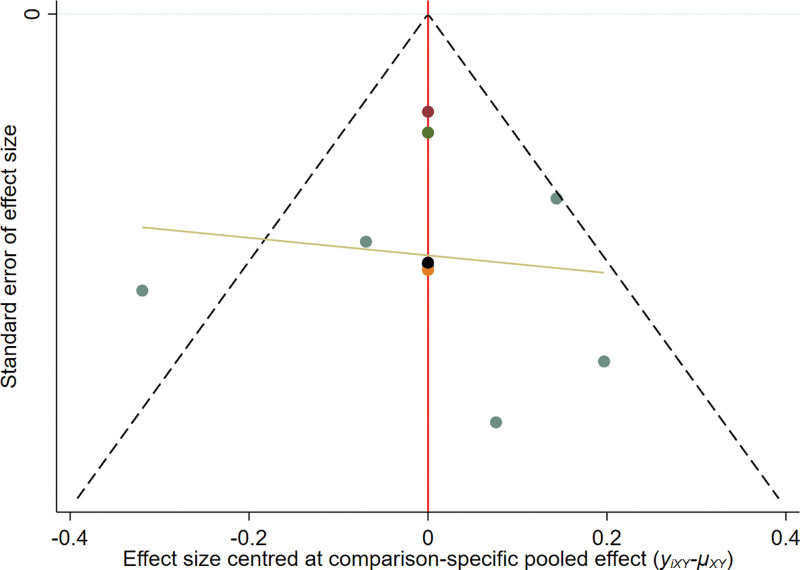
The funnel plot of DCR. DCR = disease control rate.

**Figure 7.**
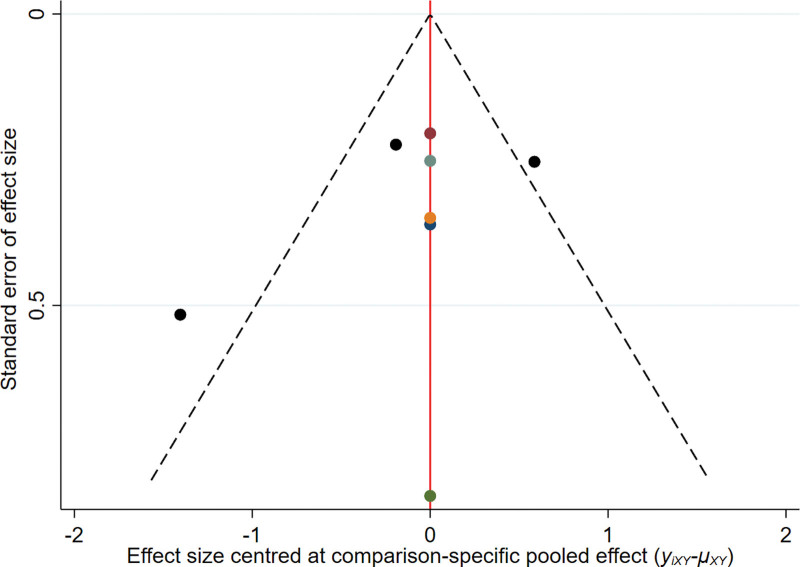
The funnel plot of OS. OS = overall survival.

From the funnel chart of objective remission rate, DCR, and OS, it can be seen that the points are scattered and incompletely symmetrical, suggesting that there may be some publication bias, and there are scattered points at the bottom of the funnel chart of each research index, indicating that there is a small sample effect (see Figs. 5 to 7).

## 4. Discussion

Breast cancer is the most common malignant tumor in women, accounting for 30% of all malignant tumors.^[23]^ In addition, it is prone to brain metastasis, second only to lung cancer, accounting for 15 to 20% of all brain metastases.^[24,25]^ In recent years, the incidence of breast cancer brain metastases (BCBM) has increased and HER2 + and Triple-negative subtypes were had a higher rate of brain metastases.^[26]^ Although significant progress has been made in the systematic treatment of BC that of BCBM is still very difficult. At present, local treatment remains the cornerstone of BCBM treatment. Organically integrating local and systemic therapies remains an urgent problem to be solved. Local treatment can destroy the blood-brain barrier, increase the concentration of drugs in the brain tissue, and better control intracranial lesions. Niwińska et al^[27,28]^ pointed out that systemic treatment after local treatment can prolong the survival time of BCBM to a great extent. Local treatment is more targeted and effective for brain metastases, but adverse reactions are more serious. Systemic therapy refers to cancer treatment methods for the whole body, including chemotherapy, endocrine therapy, immunotherapy, and targeted therapy, while therapeutic drugs can reach all parts of the body with blood circulation, thus killing tumor cells in many parts. At present, combination therapy based on whole-brain radiotherapy is becoming increasingly common in the clinic and can significantly prolong the survival time of patients.^[29]^ In addition, a variety of new drugs have shown certain curative effects on BCBM, such as mTOR inhibitor, CDK4/6 inhibitor, PARP inhibitor, so on, which brings hope to the treatment of BCBM. In this study, the efficacy of different treatment measures for BCBM was comprehensively analyzed in 14 articles, and the drugs with the best efficacy were judged according to the ranking probability of intervention drugs obtained using the Bayesian network model. The greater the probability, the better the survival and prognosis of the patients. Finally, it was concluded that WBRT + 3D-CRT + Che may be the best intervention for objective remission rate and DCR in patients with brain metastasis of BC, and WBRT + Che may be the intervention with the lowest OS risk ratio in patients with BC with brain metastasis.

In conclusion, BCBM is a systemic disease. Local treatment combined with systemic treatment can not only improve the control rate of intracranial tumors, but also improve systemic symptoms. Therefore, we should select appropriate treatment protocols based on individual conditions

## Author contributions

Conceptualization: Anhao Wu.

Methodology: Anhao Wu, Fang Zhang.

Project administration: Xin Yang, Yang Liu.

Software: Yafang Lai, Mingjian Tan.

Supervision: Zhuangqing Yang, Yafang Lai.

Writing—original draft: Anhao Wu, Fang Zhang.

Writing—review and editing: Anhao Wu, Fang Zhang, Zhuangqing Yang.
